# A packaged intervention to improve viral load monitoring within a deeply rural health district of South Africa

**DOI:** 10.1186/s12879-020-05576-5

**Published:** 2020-11-11

**Authors:** J. Brijkumar, B. A. Johnson, Y. Zhao, J. Edwards, P. Moodley, K. Pathan, S. Pillay, K. G. Castro, H. Sunpath, D. R. Kuritzkes, M. Y. S. Moosa, V. C. Marconi

**Affiliations:** 1 University of KwaZulu Natal, Nelson R Mandela School of Medicine, Durban, South Africa; 2 University of Rochester, Rochester, NY USA; 3 Emory University Rollins School of Public Health, Atlanta, GA USA; 4 School of Laboratory Medicine and Medical Sciences, National Health Laboratory Service, University of KwaZulu-Natal, Durban, South Africa; 5 Brigham and Women’s Hospital, Harvard Medical School, Boston, USA; 6 Infectious Diseases, Emory University School of Medicine, Atlanta, GA USA; 7 Emory Vaccine Center, Atlanta, USA

**Keywords:** HIV, Viral load, South Africa, Rural health, Virologic suppression, Monitoring

## Abstract

**Background:**

The KwaZulu-Natal (KZN) province of South Africa has the highest prevalence of HIV infection in the world. Viral load (VL) testing is a crucial tool for clinical and programmatic monitoring. Within uMkhanyakude district, VL suppression rates were 91% among patients with VL data; however, VL performance rates averaged only 38·7%. The objective of this study was to determine if enhanced clinic processes and community outreach could improve VL monitoring within this district.

**Methods:**

A packaged intervention was implemented at three rural clinics in the setting of the KZN HIV AIDS Drug Resistance Surveillance Study. This included file hygiene, outreach, a VL register and documentation revisions. Chart audits were used to assess fidelity. Outcome measures included percentage VL performed and suppressed. Each rural clinic was matched with a peri-urban clinic for comparison before and after the start of each phase of the intervention. Monthly sample proportions were modelled using quasi-likelihood regression methods for over-dispersed binomial data.

**Results:**

Mkuze and Jozini clinics increased VL performance overall from 33·9% and 35·3% to 75·8% and 72·4%, respectively which was significantly greater than the increases in the comparison clinics (RR 1·86 and 1·68, *p* < 0·01). VL suppression rates similarly increased overall by 39·3% and 36·2% (RR 1·84 and 1·70, *p* < 0·01). The Chart Intervention phase showed significant increases in fidelity 16 months after implementation.

**Conclusions:**

The packaged intervention improved VL performance and suppression rates overall but was significant in Mkuze and Jozini. Larger sustained efforts will be needed to have a similar impact throughout the province.

## Background

South Africa has the highest burden of human immunodeficiency virus (HIV) in the world with approximately 7·7 million people living with the disease. Therefore, South Africa, with 4.7 million (62%) people receiving antiretroviral therapy (ART), is home to the largest HIV treatment program in the world. Overall, according to United Nations Programme on HIV/AIDS (UNAIDS), the viral load (VL) suppression rate is 45% with 3·2 million of the 6·1 million people virologically suppressed [1]. The eastern coastal province of KwaZulu-Natal (KZN) experiences the highest burden of HIV infection in the country with 1·2 million people on treatment [2]. With the implementation of the national ‘universal test and treat’ programme, it is projected that nearly 6 million people nationally will be on treatment within the next few years.

With a South African national goal to end the AIDS epidemic by 2030 and with the country adopting the 90–90-90 UNAIDS goals where the third aim requires that 90% of all people on ART achieve and maintain virologic suppression [3], it is critical to optimize ART adherence for all patients in order to ensure this level of virologic suppression. Multiple research studies have validated the use of HIV-1 VL testing for monitoring ART response, determining prognosis [4–7], and identifying early virological failure which may require ART changes [8]. Therefore, VL monitoring is of critical national importance in the effort to halt HIV transmission, combat the emergence of HIV drug resistance, and decrease morbidity and mortality [9, 10].

Although the rate of virologic suppression is close to 90% for individuals having a VL, the National Health Laboratory System (NHLS) reported only 35 to 60% of patients (depending upon the facility) actually had a VL obtained within 6 months of initiation of ART [11, 12]. Viral load performance rates are low compared to VL suppression rates, and it is possible that a large percentage of those missing a VL are virologically failing or entirely out of care. Part of this disparity could be attributed to reporting, monitoring, and documenting HIV VL testing and results within the clinical setting. This study implemented a package of interventions that sought to enhance record keeping and patient outreach at several clinics within a rural KZN health district in order to determine if (a) VL monitoring could be improved and (b) the impact this change would have on virologic suppression rates.

## Methods

### Clinical sites

The packaged intervention was implemented within the context of a parent research study, The KZN HIV AIDS Drug Resistance Surveillance Study (ADReSS). ADReSS followed patients initiating first-line ART (consisting of efavirenz/tenofovir/emtricitabine) in one peri-urban and three rural clinics within KZN to determine rates of virologic failure and HIV-1 drug resistance. The peri-urban clinic was R.K. Khan Hospital (RKK) and the rural clinics were Bethesda District Hospital Clinic (CDC), Mkuze Clinic (MKC) and Jozini Clinic (JZC) all within the uMkhanyakude district. The comparison between the peri-urban and rural sites was based on the clinic population size, package of services provided, and number of equivalent staff component. Further descriptions of the sites can be found in the supplemental methods. Participants received treatment and were followed per usual standard of care by clinic staff. All patients were on the South African first line regimen (TDF/FTC/EFV). Data were analysed 6 months after starting treatment.

### Packaged intervention

**Fig. 1:**
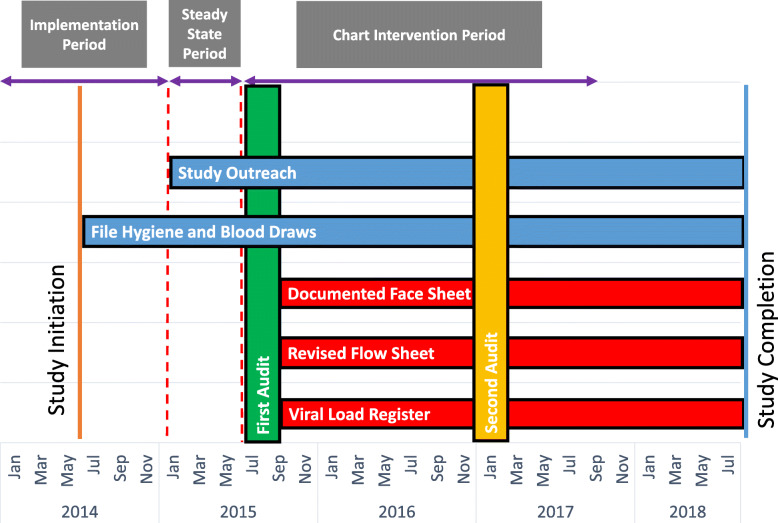
Time Line for Packaged Intervention. The first observation period, Study Implementation, began in January 2014, 6 months before initiation (yellow line) of the KwaZulu-Natal HIV Drug Resistance Surveillance Study (DReSS) and ended in December 2014 prior to when participants were due for their first HIV-1 viral load (first red dashed line). During this period, DReSS nurses assisted with clinic blood draws and maintained the study participant clinic files in a secure location. The second observation period, Study Steady State, began in January 2015 and ended in June 2015 (second red dashed line). Outreach phone calls and visits began during this period to assist in obtaining blood samples for viral loads. The final observation period, Chart Intervention, began in July 2015 at the time of the first chart audit (green bar) and ended in July 2017 several months after the second chart audit (yellow bar). This period included improvements in chart documentation and the introduction of the viral load register. DReSS completed the final follow up visit in July 2018 (blue line)

After the start of enrolment in July 2014, several process improvement phases, constituting the packaged intervention, were implemented in CDC, MKC, and JZC (Fig. 1).

#### Study implementation

Initially, the study nurses assisted the clinic in reception, filing of charts and other duties that would improve patient flow and wait times. ADReSS study nurses also assisted the clinic nurses with participant blood draws, ensured VL were obtained at the appropriate visit and kept clinic files secure for follow up visits. Frequent meetings between the principal investigator and facility management provided an opportunity to reinforce the importance of virologic monitoring.

#### Study steady state

During the course of the ADReSS follow up, it was recognized that VL monitoring within the rural clinics remained suboptimal. To address this issue, in January 2015, study staff began a campaign to call all participants within 2 weeks of their scheduled VL testing date. When, despite this, participants did not return within their VL due date, staff continued calling to encourage attendance. For those that were unable to attend clinic, blood was drawn at a convenient location via an outreach team.

#### Chart intervention

Patient clinical charts were found to be deficient in file hygiene. This involved several areas including missing and incomplete records, incorrect chronological order, and missing VL requests or results. In order to address this, the final phase was implemented starting July 2015.

- The clinical chart included a face sheet (FAS) (see supplemental section) which contained demographics elements in combination with columns for recording serial CD4 count and VL results. The FAS was often missing or incomplete. Clinic staff was instructed on how to complete the FAS. Additionally, copies of the FAS were made available at all times.
- The clinical chart also included a flow sheet (FLS) (see supplemental section) which contained two columns for documenting monthly visits. The FLS was revised (see supplemental section) to include 6 columns for the monthly visits with the sixth column uniquely shaded to emphasize whether a VL test was due on that visit.
- A VL sample log was implemented to document when a blood sample was sent to the lab for VL testing along with the outcome of the corresponding laboratory result.
- A high VL register was also implemented and contained a space for the patient demographics, contact information, elevated VL measurement, and any efforts to reach the patient for a follow-up visit. This register was designed to ensure that standard of care for repeat counselling sessions and follow-up VL were complete.

### Quality and process metric assessments

To assess the fidelity of the clinical chart documentation process, an audit was performed in July 2015, before this intervention, and in December 2016, 16 months into the Chart Intervention. The following data elements were extracted from the clinical charts of patients enrolled in the parent study: ADReSS participant identification number, clinic file number, age, gender, presence and completion of the FAS and FLS, VL requested and VL recorded.

The primary quality outcome metrics provided by the NHLS were VL suppression and VL performed. Virologic suppression was defined in the following way. Quarterly percentages of individuals having VL < 400 copies/mL were computed as weighted averages of monthly percentages of individuals with VL < 400 copies/mL, where the weights are the total number of VL tests due per month. The monthly percentages are the number of individuals with VL < 400 copies/mL in 1 month divided by the total number of VL tests due in the same month. In instances where an individual had more than 1 VL test performed in a given month, the result of last VL test was taken, so that one individual’s VL test result was counted at most once in a given month. Quarterly percentages of VL performed were defined similarly as weighted monthly averages. Here, the monthly percentage is the percent of individuals who had a VL test in a given month among those individuals who had a VL test due in the same month, and the weight is number of individuals with a VL test due that month.

Metrics from the rural clinics were compared to metrics from peri-urban clinics within the Durban Metropolitan Area as follows: CDC was compared to RKK, MKC was compared to Shallcross Clinic (SLC), and JZC was compared to Township Clinic (TSC).

### Statistical analysis

For the clinical chart audit, descriptive statistics were used to summarize the data before and after the Chart Intervention and compared using a Chi-squared test. For the VL suppression and performance analysis, descriptive statistics were used to assess data quality and explore the data within each study period. Additionally, the same data were used in the development of statistical models. The analysis allowed for the assessment of temporal trends and pre-post intervention effects through statistical modelling of aggregate sample statistics, i.e. percentages of VL suppression and percentages of VL performed.

We modelled the sample proportions of VL suppression with possible over-dispersion using quasi-likelihood methods [13]. Specifically, we modelled relative risks of outcomes by adopting a Poisson distribution for count data and logarithmic link function (see Tables 2 and 3); the robust sandwich covariance matrix was used to draw statistical inference for parameter estimates, i.e. standard errors, confidence limits. Temporal trends were summarized by modelling data over three time intervals (Fig. 1): Study Implementation period beginning in January 2014 (6 months before the study initiation) to December 2014 (just prior to when initial follow up visits began), Study Steady State period beginning January 2015 to June 2015 (just prior to the chart audit), and the Chart Intervention period starting July 2016 until July 2017. Additionally, we used a Type I error rate of 5% to reject the null hypothesis and determine that post-intervention virologic suppression or performance rates were statistically different than pre-intervention rates. All computations were performed in R version 3.4.1 (cran.r-project.org, R Core Team, 2018).

## Results

### Clinical chart documentation

In total, 23 months (excluding January 2015) of clinical chart audit data were analysed encompassing the data from 109 unique charts (Table 1). The median age (44 years) of patients at the CDC was 10–12 years older than patients at MKC and JZC, respectively; however, all treatment sites had approximately equal proportions of female patients. Substantial chart deficiencies were identified prior to the chart intervention. In particular, only 8·3% of JZC charts had documented VL test requests and 6·7% had VL results recorded. At MKZ, it was modestly better with 32·4% and 29·7%, respectively. In contrast, CDC had 83.3% of charts with VL tests requested and 75.0% VL results recorded. All percentages significantly improved after the chart intervention in both MKC and JZC. Of note, VL test requests increased to 75.7% (43.3% increase) at MKC and 26.7% (18.4% increase) at JZC. Moreover, VL results recorded increased to 54.1% (24.4% increase) at MKC and 65.0% (58.3% increase) at JZC. There were no significant changes at CDC.

**Table 1:** Chart audit results before and after the chart intervention

Characteristics	Mkuze (***n*** = 37)		Jozini (***n*** = 60)		Bethesda CDC (***n*** = 12)	
	Pre-Intervention	Post-Intervention	Pre-Intervention	Post-Intervention	Pre-Intervention	Post-Intervention
**Median Age, years (SD)**	34 (8·3)		36 (12·5)		44 (14·6)	
**Female (%)**	23 (62·2)		32 (53·3)		8 (66·7)	
**Face Sheet (%)**	29 (78·4)	36 (97·3)^b^	16 (26·7)	58 (96·7)^c^	12 (100·0)	12 (100·0)
**Flow Sheet**^**d**^ **(%)**	34 (91·9)	37 (100·0)^a^	15 (25·0)	58 (96·7)^c^	12 (100·0)	11 (91·7)
**Viral Load Test Requested (%)**	12 (32·4)	28 (75·7)^c^	5 (8·3)	16 (26·7)^b^	10 (83·3)	10 (83·3)
**Viral Load Results Recorded (%)**	11 (29·7)	20 (54·1)^b^	4 (6·7)	39 (65·0)^c^	9 (75·0)	9 (75·0)

^a^ *p* value < 0·05

^b^*p* value < 0·01

^c^ *p* value < 0·0001

^d^All Flow Sheets reviewed during the post-chart intervention audit contained the revised Flow Sheet template

### Viral load monitoring

**Fig. 2:**
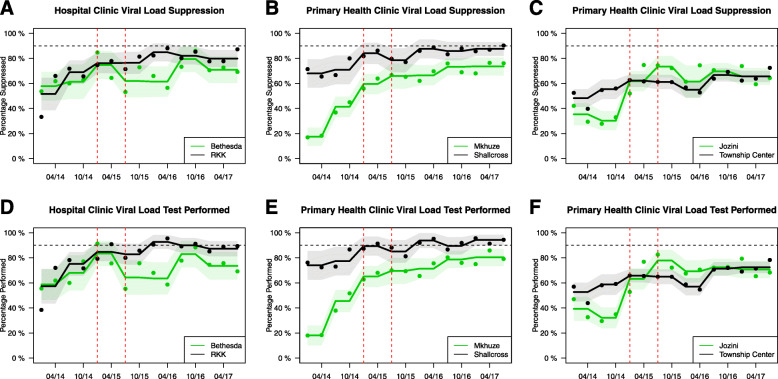
Temporal Trends in Virologic Suppression and Completion within each clinic. Virologic Suppression (**a**-**c**) and Viral Load Completion (**d**-**f**) percentages over the three phases of the study are represented for the District Hospitals, Bethesda CDC and RK Khan (**a** and **d**) and four Primary Health Clinics, Mkhuze and Shallcross (**b** and **e**) and Jozini and Township Center (**c** and **f**). Rural clinics are shown in green with the peri-urban clinics shown in black. The first period, Study Implementation, ends with the first red dashed line. The second period, Study Steady State, begins with the first red dashed line and ends with the second red dashed line. The third period, Chart Intervention, begins with the second red dashed line. The horizontal black dashed line denotes the 90% goal

During the Study Implementation period (Table 2), VL suppression rates were significantly lower for MKC and JZC (31·2% and 32·6%) compared to SLC and TSC (69·4% and 51·5%). Each of these rates increased throughout this period (Fig. 2) but were more pronounced in the rural clinics where MKC increased to 59.5% (28·3% increase) and increased to 61.8% (29·2% increase) at JZC (*p* < 0·01). The corresponding peri-urban clinics also experienced modest increases (Table 3) but the rural clinics had a substantially greater improvement (relative risk 1·57, *p* = 0·03 for MKC and 1·57, *p* < 0·01 for JZC). By the time the study reached the Steady State period, JZC had similar suppression rates as TSC (Fig. 2c) but MKC remained significantly lower than SLC (Fig. 2b) by 24·6% (*p* < 0·01). Similar trends were identified with respect to VL performance rates. MKC increased by 31·2% (from 33·9 to 65.1%) during the Study Implementation period (Fig. 2e). Likewise, JZC (Fig. 2f) increased by 27·9% (from 35·3 to 63.2%). Again, the increases in the rural clinics were significantly greater than the peri-urban clinics (RR 1·63, *p* = 0.03 and 1·51, p = 0·01 for MKC and JZC, respectively). There were no significant differences in VL Suppression or Test Performance rates between CDC and RKK during these two periods despite significant parallel increases within each site (Fig. 2a and d).

**Table 2:** Comparison of three study period values and difference of each period within and between clinics

	Site	Study Implementation Period Value	Study Steady State Period Value	Difference of Steady State and Implementation within each site (***p*** value)^d^	Chart Intervention Period Value	Difference of Intervention and Steady State within each site (***p*** value)^d^	Difference of Intervention and Implementation within each sites (***p*** value)^d^
**Viral Suppression**^**a**^	**Bethesda CDC**	59·2%	74·7%	15·5% (0·022)	68·5%^c^	−6·2% (0·42)	9·3% (0·12)
	**RK Khan (ref)**	62·3%	76·2%	13·8% (0·036)	80·9%	4·7% (0·01)	18·6% (< 0·01)
	**Mkhuze**	31·2%^c^	59·5%^c^	28·3% (< 0·01)	70·5%^c^	10·9% (< 0·01)	39·3% (< 0·01)
	**Shallcross (ref)**	69·4%	84·1%	14·7% (< 0·01)	85·4%	1·3% (0·49)	16·0% (0·04)
	**Jozini**	32·6%^c^	61·8%	29·2% (< 0·01)	68·8%^c^	7·0% (0·48)	36·2% (< 0·01)
	**Township (ref)**	51·5%	62·3%	10·8% (< 0·01)	62·9%	0·7% (0·69)	11·4% (< 0·01)
**Viral Load Test Performed**^**b**^	**Bethesda CDC**	62·5%	83·5%	21·0% (< 0·01)	71·2%^c^	−12·3% (0·05)	8·7% (0·16)
	**RK Khan (ref)**	68·4%	84·8%	16·4% (0·03)	88·3%	3·5% (0·42)	19·9% (< 0·01)
	**Mkhuze**	33·9%^c^	65·1%^c^	31·2% (< 0·01)	75·8%^c^	10·7% (< 0·01)	41·9% (< 0·01)
	**Shallcross (ref)**	75·6%	89·4%	13·8% (< 0·01)	91·3%	1·9% (0·34)	15·7% (0·08)
	**Jozini**	35·3%^c^	63·2%	27·9% (< 0·01)	72·4%^c^	9·0% (0·31)	37·1% (< 0·01)
	**Township (ref)**	55·4%	65·7%	10·3% (< 0·01)	67·5%	1·8% (0·34)	12·1% (0·01)

ed as Viral Load < 400 copies per mL/Viral Load Due

^b^Defined as Viral Load Performed/Viral Load Due

^c^Chi Square comparing sites, *p* value < 0·01

^d^Adjusted *p* value

**Table 3:** Comparison of study initiation and intervention effects between clinics

	Site	Study Initiation effect within each site	Relative-Risk between each site (***p*** value)^c^	Chart Intervention effect within each site (***p*** value)^c^	Relative-Risk between each site (***p*** value)^c^	Total effect within each site (***p*** value)^c^	Relative-Risk between each site and respective control site (***p*** value)^c^
**Viral Suppression**^**a**^	**Bethesda CDC**	15·5% (0·022)	1·03 (0·82)	−6·2% (0·42)	0·86 (0·18)	9·3% (0·12)	0·89 (0·58)
	**RK Khan (ref)**	13·8% (0·036)		4·7% (0·01)		18·6% (< 0·01)	
	**Mkhuze**	28·3% (< 0·01)	1·58 (0·03)	10·9% (< 0·01)	1·16 (< 0·01)	39·3% (< 0·01)	1·84 (< 0·01)
	**Shallcross (ref)**	14·7% (< 0·01)		1·3% (0·49)		16·0% (0·0)	
	**Jozini**	29·2% (< 0·01)	1·57 (< 0·01)	7·0% (0·48)	1·08 (0·55)	36·2% (< 0·01)	1·70 (< 0·01)
	**Township (ref)**	10·8% (< 0·01)		0·7% (0·69)		11·4% (< 0·01)	
**Viral Load Test Performed**^**b**^	**Bethesda CDC**	21·0% (< 0·01)	1·08 (0·57)	−12·3% (0·05)	0·82 (0·037)	8·7%(0·16)	0·88 (0·38)
	**RK Khan (ref)**	16·4% (0·03)		3·5% (0·42)		19·9% (< 0·01)	
	**Mkhuze**	31·2% (< 0·01)	1·63 (0·03)	10·7% (< 0·01)	1·14 (< 0·01)	41·9% (< 0·01)	1·86 (< 0·01)
	**Shallcross (ref)**	13·8% (< 0·01)		1·9% (0·34)		15·7% (0·08)	
	**Jozini**	27·9% (< 0·01)	1·51 (0·01)	9·0% (0·31)	1·11 (0·43)	37·1% (< 0·01)	1·68 (< 0·01)
	**Township (ref)**	10·3% (< 0·01)		1·8% (0·34)		12·1% (0·01)	

^a^Defined as Viral Load < 400 copies per mL/Viral Load Due

^b^Defined as Viral Load Performed/Viral Load Due

^c^Adjusted p value

At the time of the Chart Intervention period, VL Suppression was 70·5% at MKC increasing by 10·9% (*p* < 0·01) but remained below SLC (*p* < 0·01). On the other hand, JZC surpassed the VL Suppression at TSC (68·8% vs. 62·9%, *p* < 0·01). Overall, both rural clinics experienced substantial increases in VL Suppression throughout the study (39·3% and 36·2% for MKC and JZC, respectively) compared to modest increases at SLC (16·0%) and TSC (11·4%). This corresponded to a relative risk of 1·84 and 1·70 (*p* < 0·01) for the overall study observation period (Table 3) comparing MKC to SLC and JZC to TSC, respectively. Again, VL Tests Performance trends were similar with a 41·9% increase at MKC and 37·1% increase at JZC throughout the study. MKC remained significantly lower than SLC (75·8% vs. 91·3%) while JZC was significantly higher than TSC (72·4% vs. 67·5%). The VL Suppression and Test Performance rates significantly declined at CDC during this period becoming lower than RKK (*p* < 0·01).

## Discussion

The majority of reports on HIV clinical outcomes from sub-Saharan Africa are derived from large, well-resourced, urban clinics. As a result, there are large deficiencies in our understanding of the epidemic across entire regions. This report is the first description of actual conditions within one of the most heavily burdened, rural health districts in the world. Despite worrisome deficiencies in virologic monitoring from 2014, implementation of a site-directed package of interventions resulted in significant increases in VL suppression and completion rates that were sustained over the ensuing 3 years. Although most of the clinics evaluated in this report remained below the 90% goal in 2017, continued similar efforts may eventually close the gap.

Data currently show that HIV drug resistance in South Africa is approaching a tipping point with the prevalence of known pre-treatment drug resistance (PDR) estimated at well over 10% [14, 15]. Because HIV drug resistance is associated with virologic failure, increased HIV transmission, and mortality, VL monitoring and suppression are critical goals for the national program. In order to address these goals, South African healthcare providers must identify the full scope of virologic failure in both their clinic and region; however, without enhanced documentation and effective community outreach, such information will remain incomplete. The collection of data and its utilization for clinical and public health purposes are the bedrock of quality healthcare.

There are multiple factors that drive missing VL results including clinic deficiencies, laboratory errors and patient barriers [16]. An initial root-cause analysis of the clinics in this health district identified several areas that required attention. For example, the clinical chart documentation was improperly completed (if at all), medication prescription and duration of medication supply information was often missing, and for many charts, the only documentation of a patient visit was the words “for refill buya [‘to return’ in isiZulu] one month”. Orders requesting VL testing on the appropriate month were missing, and among those who were tested, the majority of charts did not record the VL test results. These scenarios are especially common in critically understaffed and overburdened rural clinics where nurses are multi-tasking and expected to manage several health programs. Tedious, albeit important, documentation is often a convenient sacrifice to accommodate other more urgent tasks [17]. Additionally, it was discovered that family members not infrequently collected medication for patients precluding their ability to have VL testing performed. Finally, lost specimens or technical issues could arise within the laboratory.

There were several aspects of the packaged intervention that could account for the increases in VL monitoring observed in MKC and JZC above the increases observed in their peri-urban counterparts. The introduction of highly trained study nurses into the clinic along with periodic research doctor education for clinic nurses and staff during the Study Implementation period could have had the greatest impact as the most dramatic increase occurred during this time. Additionally, outreach during the Steady State period was effective at identifying and obtaining blood samples from patients who were due or overdue. Finally, a concerted effort to improve documentation during the Chart Intervention period allowed the increases to be sustained over time. These changes were a direct result of a series of collaborative meetings between clinic management and the ADReSS team. These meetings resulted in policy changes at the level of the health district including the introduction of the Viral Load Register and revised FLS. It was evident that a major benefit of the new form was shading one of the columns to “nudge” nurses and doctors for when a VL was due. The district managers also established ward-based outreach teams and community care givers to extend the efforts of the study nurses to the entire clinic population.

Although the peri-urban clinics did not implement the packaged intervention, the significant increase in virologic monitoring that was observed in these sites could be attributed to improved staff-to-patient ratios, assistance from nongovernmental organization partners, better use and monitoring of the VL sample log, easier and more accessible modes of transport, closer monitoring of individuals failing treatment, and tracking patients who fell out of care. The rural district hospital clinic, CDC, already approximated the peri-urban counterpart and did not change much over time. The fact that CDC is smaller and focused on more complicated patients with closer monitoring than in the peripheral primary health clinics could explain this phenomenon.

There were two broader programs which began after the Chart Intervention period that could have also improved virologic monitoring for the rural health clinics. In the first program, patients who were virologically suppressed were put on the Chronic Care Medicines Dispensing and Distribution programme. This is a public-private partnership where patients could collect their medication from private pharmacies at their convenience. Similarly, adherence clubs were established so that patients who were virologically suppressed would assemble at a convenient location close to their home (e.g. local community halls or places of worship) to interact and encourage adherence as well as to collect pre-packaged medications. Both options rewarded optimal adherence and simultaneously helped to reduce the queue in the clinics. In addition to benefiting patients with a convenient pick-up location, this and newer triage algorithms have helped to decompress clinic workload. Other strategies that may assist in reaching the 90 90 90 goals include Fast Queues for virologically suppressed patients, frequent workshops and training of staff on early warning indicators for treatment failure, and appointing a VL champion to maintain and monitor the VL register.

### Study limitations

Because this intervention was implemented in a deeply rural area of KZN, the findings may not apply to all clinics that provide ART in similar low and middle income settings. Additionally, this study was not a randomised trial so any comparisons with the control district may not reflect a balance of important covariates functioning at these different locations. Finally, the audit did not assess every patient file in the clinic but focused instead on patients involved in the observational parent study. This could have led to a bias in the implementation of the file component of the intervention. The intervention and audit also intersected cross-sectionally in the clinic such that new patients enrolled in the clinic could be impacted differently than those who had been attending the clinic for several years. Another limitation of this study is that the parent study ended in July 2018 hence the observation and follow up period also ended. Current data may differ due to programmatic and treatment guideline changes.

## Conclusions

Substantial improvements in virologic monitoring and suppression occurred following implementation of a packaged intervention designed to improve care of persons living with HIV in a rural health district of KZN. Some of these improvements may have resulted from greater attention to VL monitoring in the region as a whole. The greatest change occurred at the earliest phase of this intervention and is likely related to enhanced attention to and focus on obtaining a VL at the appropriate time. Minor adjustments to clinical documentation, targeted outreach, and the introduction of a VL register could have contributed to the sustainability of this change. These findings demonstrate the well-described benefits that research endeavours provide to clinical programs. South Africa, with one of the largest ARV programmes in the world, would not be able to sustain the programme if accurate, reliable and verifiable data are not recorded. With the ever decreasing budget from the conditional grant, the South African government in conjunction with Presidents Emergency Plan For AIDS Relief (PEPFAR) has had to leverage the limited resources available and incorporate the assistance of outreach teams in order to combat the HIV epidemic in South Africa. Focus should also be placed on accurate documentation and data capture using periodic audits to identify which clinics need support and enhanced instruction. Larger sustained efforts will be needed to substantively impact these metrics while remaining cost-effective. Only, then will it be possible to assess exactly where our challenges lie in combating this epidemic.

## Supplementary Information

**Additional file 1: Supplementary Methods**. **Annexure 1**. Department of Health Face Sheet. **Annexure 2**. Original Department of Health Flow Sheet. **Annexure 3**. Revised Flow Sheet.

## Data Availability

The data that support the findings of this study are available from the National Health Laboratory Service in South Africa but restrictions apply to the availability of these data, which were used under license for the current study, and so are not publicly available. Data are however available from the authors upon reasonable request and with permission of National Health Laboratory Service in South Africa.

## Abbreviations

KZN: KwaZulu-Natal
VL: viral load
ART: Antiretroviral therapy
HIV: Human immunodeficiency virus
AIDS: Acquired Immunodefiency syndrome
UNAIDS: United Nations Programme on HIV and AIDS
NHLS: National health laboratory services
ADReSS: AIDS Drug Resistance Surveillance Study
RKK: R K Khan Hospital
CDC: Bethesda district hospital clinic
MKC: Mkhuze clinic
JZC: Jozini clinic
FAS: face sheet
FLS: flow sheet
SLC: Shallcross clinic
TSC: Township clinic
PDR: pre-treatment drug resistance
PEPFAR: Presidents Emergency Plan For AIDS Relief

## References

[1] UNAIDS Data 2017. Geneva, Switzerland: Joint United Nations Programme on HIV/AIDS (UNAIDS);2017.

[2] HIV/AIDS/STI Dashboard Indicators National Feedback Per Province: 2016/17 Financial year quarter 2. District Health Information System, Department of Health Republic of South Africa; December 1, 2016 2016.

[3] Analysis of Big Data for Better Targeting of ART Adherence Strategies: Spatial Clustering Analysis of Viral Load Suppression by South African Province, District, Sub-District and Facility (April 2014–March 2015). Washington, DC: World Bank; 2015.

[4] Coombs RW, Welles SL, Hooper C (1996). Association of plasma human immunodeficiency virus type 1 RNA level with risk of clinical progression in patients with advanced infection. AIDS Clinical Trials Group (ACTG) 116B/117 study team. ACTG virology Committee resistance and HIV-1 RNA working groups. J Infect Dis.

[5] Gazzard B, Committee BW (2005). British HIV Association (BHIVA) guidelines for the treatment of HIV-infected adults with antiretroviral therapy (2005). HIV Med.

[6] Mellors JW, Rinaldo CR, Gupta P, White RM, Todd JA, Kingsley LA (1996). Prognosis in HIV-1 infection predicted by the quantity of virus in plasma. Science..

[7] O'Brien WA, Hartigan PM, Daar ES, Simberkoff MS, Hamilton JD (1997). Changes in plasma HIV RNA levels and CD4+ lymphocyte counts predict both response to antiretroviral therapy and therapeutic failure. VA cooperative study group on AIDS. Ann Intern Med.

[8] In: nd, ed. Consolidated Guidelines on the Use of Antiretroviral Drugs for Treating and Preventing HIV Infection: Recommendations for a Public Health Approach. Geneva 2016. 27466667

[9] Abbas UL, Anderson RM, Mellors JW (2006). Potential impact of antiretroviral therapy on HIV-1 transmission and AIDS mortality in resource-limited settings. J Acquir Immune Defic Syndr.

[10] Tanser F, Barnighausen T, Grapsa E, Zaidi J, Newell ML (2013). High coverage of ART associated with decline in risk of HIV acquisition in rural KwaZulu-Natal, South Africa. Science.

[11] South Africa Global AIDS Response Progress Report (GARPR) 2015. Pretoria, South Africa: South African National AIDS Council (SANAC); 2015.

[12] HIV/AIDS/STI Dashboard Indicators National Feedback Per Province: 2016/17 Financial year quarter 2*.* District Health Information System; 01 December 2016.

[13] McCullagh P, Nelder JA. Generalized linear models. London ; New York: Chapman and Hall; 1983.

[14] Chimukangara B, Kharsany ABM, Lessells RJ (2019). Moderate-to-high levels of pretreatment HIV drug resistance in KwaZulu-Natal Province, South Africa. AIDS Res Hum Retroviruses.

[15] District Health Plan 2015/2016*:* Umkhanyakude District*,* Kwazulu-Natal. 2015.

[16] Mutenda N, Bukowski A, Nitschke AM (2016). Assessment of the World Health Organization's HIV drug resistance early warning indicators in Main and decentralized outreach antiretroviral therapy sites in Namibia. PLoS One.

[17] Pham MD, Romero L, Parnell B, Anderson DA, Crowe SM, Luchters S (2017). Feasibility of antiretroviral treatment monitoring in the era of decentralized HIV care: a systematic review. AIDS Res Ther.

