# Supplementary material for: A packaged intervention to improve viral load monitoring within a deeply rural health district of South Africa
Source: BMC Infect Dis. 2020 Nov 11;20:836. doi: 10.1186/s12879-020-05576-5 (PMC7659110; doi:10.1186/s12879-020-05576-5)
Supplement: Supplementary file 1 — Additional file 1: Supplementary Methods. Annexure 1. Department of Health Face Sheet. Annexure 2. Original Department of Health Flow Sheet. Annexure 3. Revised Flow Sheet. [file 12879_2020_5576_MOESM1_ESM.docx]

**Supplementary Methods**

*Study Setting*

The district of uMkhanyakude (UMK) is the northern-most district in KwaZulu-Natal (KZN) and has a population of 638,011 with an estimated HIV prevalence of 29.4%. It shares a border with Mozambique and Swaziland. UMK is deeply rural, the most sparsely populated district, and ranked number 51 of the 52 most deprived districts in the country. Only 5% of the roads are surfaced and the distances for patients to travel to the clinics are extensive. For this analysis, we assessed the Bethesda District Hospital Clinic (CDC) which is situated within the hospital, Mkuze Clinic (MKC) and Jozini Clinic (JZC). The CDC is situated at the summit of the Ubombo Mountain and has five nurses caring for over 1523 HIV positive patients. It is also the referral centre for JZC and MKC. JZC is a primary health clinic (PHC), is open 24 hours, and has five nurses caring for 2839 HIV positive patients including individuals from neighbouring countries. MKC is a PHC staffed with five nurses caring for 2574 HIV positive patients. Doctors based at CDC provide support to the antiretroviral clinic at CDC and visit MKC and JZC on a rotational basis. These clinics were chosen to represent the diversity of clinical operations within the district and were clinics participating in the KZN HIV AIDS Drug Resistance Surveillance Study (ADReSS). ADReSS was an observational assessment of antiretroviral (ART) response and HIV drug resistance. We assessed quality and process metrics before and after two phases of the packaged intervention from these clinics. These results were then compared to similar clinics in a metropolitan district within KZN in order to determine the potential effect of the packaged intervention. These clinics were RK Khan (RKK), Shallcross (SLC) and Township (TSC). RKK was compared with CDC, SLC compared with MKC, and TSC compared with LZC. Similar to BTD, the HIV clinic at RK Khan Hospital is situated within the hospital while SLC and TSC are PHC located within close proximity. These clinics also refer patients to RKK. The patients at RKK are fully managed by doctors and have 4600 active HIV positive patients. SLC cares for 2000 HIV positive patients, and TSC cares for 5000 HIV positive patients. Patients from both clinics are managed by nurses.

*Interventions at the Clinic Level*

A packaged intervention was implemented at the three clinic sites with permission from the UMK District office and the Medical Manager of Bethesda Hospital who is responsible for all district clinics. The Department of Health (DoH) has adequate tools for data recording but consistent monitoring of proper data recording has been insufficient. Additionally, most of the lay counsellors in DOH clinics were reassigned to other positions outside of the clinics for budgetary purposes thus putting an added strain on the existing nursing resources. This has led to frequently missed counselling sessions where the importance of viral load (VL) monitoring could be addressed with patients. Additionally, there have been missed opportunities to request VL testing and capture VL results for entering into the medical chart. As a result of these deficiencies, each clinic was directed by the UMK district health manager to initiate the following strategies in July 2015^[1]^: (1) proper utilization of the medical chart face sheet (FAS), (2) insert and maintain a revised follow-up flow sheet (FLS) in the chart, (3) implement a VL sample log, and (4) high VL register. The FLS was a template form that is typically completed at each visit and contains the visit date, weight, tuberculosis and pregnancy questions, current ART regimen, clinical staging, and the latest VL measurement and date (completed only for that visit column). The revised FLS had a shaded column added to all the above to record the next VL due date and an additional column to record the result. This shaded column served as a prompt which would “nudge” the nurse or doctor to request the VL during the present visit. The VL sample log contained a space for a bar code representing the tube of blood drawn for the VL, the date of the blood draw, and the final VL result. The high VL register contained a space for the patient demographics, contact information, elevated VL measurement, and any efforts to reach the patient for a follow-up visit. This register was designed to ensure that standard of care for repeat counselling sessions and follow-up VL were complete.

*Interventions at the District Level*

Simultaneous to the clinic interventions, UMK initiated several programmes district-wide to improve clinic retention and reduce clinic workload. With universal test and treat, the volume of patients initiating ART has risen dramatically. In order to decongest the hospital and clinics, multiple interventions were adopted for the district starting in July 2015 which included (1) a VL anniversary, (2) Adherence Clubs, (3) the Centralized Chronic Medication Dispensing and Distribution (CCMDD) programme, and (4) Community Care Givers (CCG) and Ward-Based Outreach Teams (WBOT). Greater awareness was created around VL monitoring thus VL anniversaries became a strategy to ensure VL tests will be requested at the appropriate time. Adherence Clubs were also implemented in order to decongest clinics and hospitals. These were areas outside the hospital where patients could access basic blood pressure measurements and blood glucose monitoring while interacting with peers. Venues included community halls, temples, and churches where patients could meet and collect their medication pre-packed by the hospital. This was an opportunity for patients to openly discuss challenges with peers. Within the CCMDD programme, patients who were virologically suppressed had an opportunity to collect ART from private retail pharmacies, local meeting halls, or houses of worship. This programme greatly reduced the amount of foot traffic in the individual clinics.^[2, 3]^ Finally, CCG and WBOT were employed by the district to track patients who had been lost to care so that they could be brought back into care for treatment and VL monitoring. As an appreciated and effective outreach effort within their communities, high VL suppression rates have been observed with the use of CCG and WBOT in South Africa.^[2, 3]^

*Laboratory Measures*

Five NHLS laboratories perform HIV-1 VL testing in KZN. These are based at Ngwelezane Hospital, Madadeni Hospital, Edendale Hospital, Addington Hospital, and Inkosi Albert Luthuli Central Hospital (IALCH). The Virologists at IALCH provided quality assurance outreach to the Ngwelezane Hospital Laboratory which performed VL testing for uMkhanyakude (UMK). HIV-1 VL testing was performed using the Cobas Ampliprep-Cobas TaqMan System (Roche Diagnostics, Munich, Germany). All test results from this system are integrated with the NHLS laboratory information system (TrakCare, Intersystems, Australia) and are then archived in a central data warehouse in the NHLS IT Department in Sandringham, Johannesburg. Patient results are downloaded in spreadsheet format according to the NHLS IT security policy. Monthly VL quality metrics, number of tests <400 copies/mL, and total tests performed from the three UMK clinics were available from January 2015 until January 2017. In parallel, similar quality metrics were available from the three peri-urban clinics.

**References**

1. Kekana MM C-HS, Mongwe MW, Railton J, Peters RPH. **Quality improvement of the viral load programme in Mopani District, Limpopo Province**. *HIV Nursing Matters*:35-39.

2. Marcus TS, Hugo J, Jinabhai CC. **Which primary care model? A qualitative analysis of ward-based outreach teams in South Africa**. *Afr J Prim Health Care Fam Med* 2017; 9(1):e1-e8.

3. Khuzwayo LS, Moshabela M. **The perceived role of ward-based primary healthcare outreach teams in rural KwaZulu-Natal, South Africa**. *Afr J Prim Health Care Fam Med* 2017; 9(1):e1-e5.

Annexure1. Department of Health Face Sheet.

Annexure 2. Original Department of Health Flow Sheet.

Annexure 3. Revised Flow Sheet.
